# Lithium-Associated Kidney Microcysts

**DOI:** 10.1100/tsw.2008.112

**Published:** 2008-08-31

**Authors:** Jennifer Tuazon, David Casalino, Ehteshamuddin Syed, Daniel Batlle

**Affiliations:** ^1^ Division of Nephrology and Hypertension, Northwestern University Feinberg School of Medicine, Chicago, USA; ^2^ Division of Radiology Northwestern, University Feinberg School of Medicine, Chicago, USA

**Keywords:** lithium, kidney disease, nephropathy, cysts, MRI

## Abstract

Long-term lithium therapy is associated with impairment in concentrating ability and, occasionally, progression to advanced chronic kidney disease from tubulointerstitial nephropathy. Biopsy findings in patients with lithium-induced chronic tubulointerstitial nephropathy include tubular atrophy and interstitial fibrosis interspersed with tubular cysts and dilatations. Recent studies have shown that cysts are seen in 33–62.5% of the patients undergoing lithium therapy. MR imaging is highly capable of defining renal morphological features and has been demonstrated to be superior to US and CT scan for the visualization of small renal cysts. The microcysts are found in both cortex and medulla, particularly in the regions with extensive atrophy and fibrosis, and can be multiple and bilateral. They tend to be sparse and do not normally exceed 12 mm in diameter. The renal microcysts in the image here reported are subtle, but consistent with lithium-induced chronic nephropathy. An MRI of the kidneys provides noninvasive evidence that strengthens the diagnosis of lithium-induced nephropathy.

